# Supplementary material for: Micronutrient status and dietary patterns among children with autism in Central Vietnam: A cross-sectional baseline survey to inform targeted intervention
Source: PLOS Glob Public Health. 2026 May 13;6(5):e0006385. doi: 10.1371/journal.pgph.0006385 (PMC13170880; doi:10.1371/journal.pgph.0006385)
Supplement: S1 File — This file details the methodology and formulas used for sample size estimation and power analysis. (DOCX) [file pgph.0006385.s001.docx]

**S1 File. Detailed Statistical Power and Precision Calculations**

**Sample size and power**

All eligible children aged 2–9 years attending the five participating centres during the study period were invited to participate. A total of 48 children met the inclusion criteria, and all consented to participate, resulting in 100% enrolment among eligible participants at these centres. Consequently, this study employed a census-based approach rather than a priori sample size calculation [1].

Given the absence of a provincial registry of children with ASD and the exploratory nature of the study, this approach was chosen to maximize data completeness and minimize selection bias within participating centres.

**Precision for prevalence estimates**

For a single proportion p the half-width (HW) of the 95 % confidence interval is [1]:

$HW=1.96\times\surd\frac{p(1-p)}{n}$ (1)

With p = 0.40 (median zinc-inadequacy prevalence reported by Sharp et al. 2013 [2]) and n = 48, the half-width is 0.14; thus a prevalence of 40 % can be estimated with a confidence interval of 26 %–54 %, which is acceptable for a small descriptive survey.

**Power for age-group comparisons**

The final sample comprised 25 preschool children (< 5 years) and 23 school-age children (5–9 years). With these group sizes a two-sided test at α = 0.05, retains 80 % power to detect a risk difference of ≥ 35 percentage points in nutrient inadequacy. For continuous outcomes the same allocation provides 80 % power to detect an effect size of 0.7 standard deviations using the Mann–Whitney test.

**References for S1 File**

1. Dicker RC, Coronado F, Koo D, Parrish RG. Principles of epidemiology in public health practice; an introduction to applied epidemiology and biostatistics. 3rd ed. [cited 1 July 2025]. Available: https://stacks.cdc.gov/view/cdc/6914

2. Sharp WG, Berry RC, McCracken C, Nuhu NN, Marvel E, Saulnier CA, et al. Feeding Problems and Nutrient Intake in Children with Autism Spectrum Disorders: A Meta-analysis and Comprehensive Review of the Literature. J Autism Dev Disord. 2013;43: 2159–2173. doi:10.1007/s10803-013-1771-5
