# Supplementary material for: Micronutrient status and dietary patterns among children with autism in Central Vietnam: A cross-sectional baseline survey to inform targeted intervention
Source: PLOS Glob Public Health. 2026 May 13;6(5):e0006385. doi: 10.1371/journal.pgph.0006385 (PMC13170880; doi:10.1371/journal.pgph.0006385)
Supplement: S2 File — This form was used to collect detailed information on the types and quantities of foods and beverages consumed by the child during the previous day. (DOCX) [file pgph.0006385.s002.docx]

**S2 File. 24-Hour Dietary Recall Form**

**Participant ID:** __________
**Interview Date:** ___ / ___ / ___
**Interviewer Name:** ____________________
**Interview Start Time:** ________
**Setting:** ☐ Home ☐ Intervention center ☐ Other: ___________
**Caregiver Respondent:** ☐ Mother ☐ Father ☐ Grandparent ☐ Other: ___________

| **Time of Consumption** | **Dish Name** | **Food Item** | **Unit** | **Amount Consumed** | **Amount Left Uneaten** | **Cooked Weight (g)** | **Raw Weight (g)** | **Food Code** |
| --- | --- | --- | --- | --- | --- | --- | --- | --- |
|  |  |  |  |  |  |  |  |  |
|  |  |  |  |  |  |  |  |  |
|  |  |  |  |  |  |  |  |  |
|  |  |  |  |  |  |  |  |  |
|  |  |  |  |  |  |  |  |  |
|  |  |  |  |  |  |  |  |  |
