# Supplementary material for: Micronutrient status and dietary patterns among children with autism in Central Vietnam: A cross-sectional baseline survey to inform targeted intervention
Source: PLOS Glob Public Health. 2026 May 13;6(5):e0006385. doi: 10.1371/journal.pgph.0006385 (PMC13170880; doi:10.1371/journal.pgph.0006385)
Supplement: S3 File — Semi-quantitative food frequency questionnaire used to assess habitual intake of eight core food groups over the past month. (DOCX) [file pgph.0006385.s003.docx]

**S3 File. Food Frequency Questionnaire**

| **Food Group** | **Daily** | **3–4 times/week** | **1–2 times/week** | **1–2 times/month** | **Rarely** | **Never** |
| --- | --- | --- | --- | --- | --- | --- |
| Group 1: Staple foods (rice, sweet potatoes, maize) |  |  |  |  |  |  |
| Group 2: Legumes and nuts (soybeans, green beans, sesame seeds, peanuts) |  |  |  |  |  |  |
| Group 3: Milk and dairy products |  |  |  |  |  |  |
| Group 4: Meats, fish, and seafood |  |  |  |  |  |  |
| Group 5: Eggs and egg products |  |  |  |  |  |  |
| Group 6: Yellow/orange vegetables and dark green leafy vegetables |  |  |  |  |  |  |
| Group 7: Other vegetables and tubers (e.g., chayote) |  |  |  |  |  |  |
| Group 8: Cooking oils and animal fats |  |  |  |  |  |  |
