# Supplementary material for: Micronutrient status and dietary patterns among children with autism in Central Vietnam: A cross-sectional baseline survey to inform targeted intervention
Source: PLOS Glob Public Health. 2026 May 13;6(5):e0006385. doi: 10.1371/journal.pgph.0006385 (PMC13170880; doi:10.1371/journal.pgph.0006385)
Supplement: S1 Table — Comparison of inadequate intake prevalence across age groups with risk difference and 95% confidence intervals. (DOCX) [file pgph.0006385.s004.docx]

S1 Table. Prevalence of nutrient inadequacy by age group and risk difference (*n* = 48)

| **Nutrient** | **<5 years (n=25)** | **5-9 years (n=23)** | **Risk difference (95% CI)** | **p-value** |
| --- | --- | --- | --- | --- |
| Fiber | 25/25 (100.0) | 23/23 (100.0) | NA | NA |
| Iodine | 24/25 (96.0) | 23/23 (100.0) | -4.0 (-11.7; 3.7) | >0.99 |
| Zinc | 14/25 (56.0) | 17/23 (73.9) | -17.9 (-44.3; 8.5) | 0.321 |
| Vitamin A | 12/25 (48.0) | 16/23 (69.6) | -21.6 (-48.7; 5.5) | 0.212 |
| Vitamin C | 15/25 (60.0) | 16/23 (69.6) | -9.6 (-36.4; 17.2) | 0.641 |
| Postassium | 18/25 (72.0) | 21/23 (91.3) | -19.3 (-40.3; 1.7) | 0.140 |
| **Calcium** | **10/25 (40.0)** | **17/23 (73.9)** | **-33.9 (-60.1; -7.6)** | **0.037** |
| Vitamin PP | 6/25 (24.0) | 12/23 (52.2) | -28.2 (-54.6; -1.7) | 0.145 |
| Magnesium | 6/25 (24.0) | 12/23 (52.2) | -28.2 (-54.6; -1.7) | 0.153 |
| Phosphorus | 7/25 (28.0) | 10/23 (43.5) | -15.5 (-42.3; 11.3) | 0.386 |
| Sodium (excess) | 13/25 (52.0) | 10/23 (43.5) | 8.5 (-19.6; 36.6) | 0.587 |
| Iron (10%) | 7/25 (28.0) | 5/23 (21.7) | 6.3 (-18.1; 30.6) | 0.027 |
| Copper | 5/25 (20.0) | 5/23 (21.7) | -1.7 (-24.7; 21.3) | 0.101 |
| Vitamin B2 | 5/25 (20.0) | 12/23 (52.2) | -32.2 (-57.9; -6.5) | 0.052 |
| Vitamin B1 | 4/25 (16.0) | 6/23 (26.1) | -10.1 (-33.1; 12.8) | 0.646 |
| Selenium | 4/25 (16.0) | 2/23 (8.7) | 7.3 (-11.1; 25.7) | 0.783 |

*Risk difference (RD) and 95% confidence intervals (CI).*

*Nutrient inadequacy was defined as daily intake <75% of the Vietnamese Recommended Dietary Allowance (RDA, 2016).*

Children aged 5–9 years had a significantly higher prevalence of inadequate calcium intake than those <5 years (73.9% vs. 40.0%; RD: –33.9 [–60.1; –7.6], *p* = 0.037). Similar trends were observed for vitamin B2 and magnesium, although not statistically significant. Complete data are provided in Supplementary Table S1.
