# Supplementary material for: Micronutrient status and dietary patterns among children with autism in Central Vietnam: A cross-sectional baseline survey to inform targeted intervention
Source: PLOS Glob Public Health. 2026 May 13;6(5):e0006385. doi: 10.1371/journal.pgph.0006385 (PMC13170880; doi:10.1371/journal.pgph.0006385)
Supplement: S2 Table — Distribution of habitual intake frequencies across eight food groups, categorized by six consumption levels. (DOCX) [file pgph.0006385.s005.docx]

S2 Table. Frequency of food group consumption among children with ASD (*n* = 48)

| **Food group** | **Daily/almost daily** | **Weekly (1-4 times/week)** | **Rarely/never** |
| --- | --- | --- | --- |
| Cereals and starchy staples | 48 (100.0) | NA | NA |
| Legumes, seeds, and nuts | 7 (14.6) | 19 (39.6) | 22 (45.9) |
| Milk and dairy products | 37 (77.1) | 6 (12.5) | 5 (10.4) |
| Meat, fish, and seafood | 38 (79.2) | 8 (16.7) | 2 (4.2) |
| Eggs and egg products | 27 (56.2) | 21 (43.8) | NA |
| Dark green leafy and orange vegetables | 33 (68.8) | 9 (18.8) | 6 (12.6) |
| Other vegetables (e.g., chayote, cabbage) | 6 (12.5) | 14 (29.2) | 28 (58.3) |
| Cooking oil and fats | 42 (87.6) | 3 (6.3) | 3 (6.3) |

*NA: Not applicable.*

*Values are n (% of 48). “Daily / almost daily” = ≥ 5 times per week.*

Food frequency analysis showed all children consumed cereals daily. However, 45.9% rarely or never consumed legumes and nuts, 58.3% consumed few other vegetables, and 22.9% consumed dairy infrequently. These patterns may explain the high prevalence of micronutrient inadequacy. Details are presented in Supplementary Table S2.
