# Supplementary material for: Micronutrient status and dietary patterns among children with autism in Central Vietnam: A cross-sectional baseline survey to inform targeted intervention
Source: PLOS Glob Public Health. 2026 May 13;6(5):e0006385. doi: 10.1371/journal.pgph.0006385 (PMC13170880; doi:10.1371/journal.pgph.0006385)
Supplement: Attachment — Submitted filename: Response to Reviewers.docx [file pgph.0006385.s007.docx]

**Micronutrient status and dietary patterns among children with autism in Central Vietnam: A cross-sectional baseline survey to inform targeted intervention**

*Manuscript Number: PGPH-D-25-01847*

We take this opportunity to thank the editor and reviewers of our paper for their kind collaboration to the improvement of this manuscript. We have taken intro account all the concerns raised and we have made the suggested modifications. We have implemented numerous improvements to the paper. Below we justify our replies to the suggestions made by the repected reviewers of the paper.

| Editor and reviewers comments | Author(s) response |
| --- | --- |
| Introduction needs restructuring  The current introduction presents useful background but does not clearly articulate:  -The specific research gap,  -The problem statement,  -Why this study is needed in Central Vietnam, and  -How this study builds on and differs from existing Vietnamese literature.  Consider ending the introduction with:  -A concise summary of known gaps,  -A clear statement of objectives or research questions.  Absence of explicit study objectives  Your introduction does not explicitly state what the study set out to measure. Please add specific, numbered objectives such as:  -To assess anthropometric nutritional status…  -To quantify micronutrient biomarkers…  -To describe dietary intake patterns…  -To compare preschool vs. school-aged children…  This will improve alignment between introduction, results, and discussion. | We thank the editor for this constructive comment, which has helped us substantially improve the clarity and structure of the Introduction.  Research Gap and Problem Statement: We have explicitly articulated the lack of integrated data linking anthropometry, dietary intake, and micronutrient biomarkers in Vietnam, particularly in regional provinces like Nghe An.  Regional Context: We have added socio-economic context for Central Vietnam, highlighting the disparities between this region and metropolitan economic centers (e.g., lower per capita income and limited specialized medical services) to justify why this study is localized in Nghe An.  Relationship to Existing Literature: We have clarified how this study builds upon previous anthropometric research by adding biochemical and dietary components to create a more holistic nutritional profile.  Explicit Objectives: As suggested, we have added a numbered list of four specific objectives at the end of the Introduction to clarify the study’s scope and improve alignment with the Results and Discussion sections.  (See Introduction, page 4) |
| Sampling and study design need clearer description  Reviewer 1’s concern is valid. The manuscript currently does not explain:  -How the five centres were selected (all centres in the province? convenience? purposive selection?).  -Whether the sample is representative of ASD children in the province.  -How many children in total attend these centres, and whether the included 48 reflect 100% enrolment.  -Even with a census approach, transparency is necessary. | We thank the Editor and Reviewer for this important comment regarding sampling and study design transparency. We agree that clearer description is necessary and have revised the manuscript accordingly.  Centre Selection: We have clarified that the five centres were selected using a convenience-based participatory approach. All identifiable and accessible centres in Nghe An province were formally invited, and five provided institutional approval to participate (See Section Design, population, and settings, Page 5).  Sampling Approach: To ensure transparency, we have explicitly stated that this study employed a census-based approach. We invited the total eligible population (48 children) enrolled at these five facilities who met our strict inclusion criteria.  Enrolment Rate: The final sample of n = 48 reflects a 100% participation rate of the eligible children present at these sites during the survey period (See Section Sample Size, Page 6).  Representativeness: While the sample size is modest, these five centres are among the primary providers of ASD services in the province, covering both urban and rural catchment areas. We have acknowledged the exploratory nature of the study while providing detailed power calculations in S1 File to justify the current sample for age-group comparisons. |
| Operational definitions missing  Please define:  -Constipation  -Diarrhea  -Inadequate intake (<75% RDA) and explain why this cut-off was chosen  -Low serum zinc thresholds (child age-specific?)  -Overweight/obesity (for 5–9 years, clarify z-score cut-off)  Operational definitions help improve reproducibility. | We thank the Editor for highlighting the need for clearer operational definitions to improve reproducibility. We have revised the Methods section accordingly and now explicitly define all requested variables as follows:  Constipation was defined as fewer than three bowel movements per week and/or the passage of hard or difficult-to-pass stools during the past month.  Diarrhoea is defined as the passage of 3 or more loose or liquid stools per day (or more frequent passage than is normal for the individual).  Inadequate dietary intake: Operationally defined as <75% of the age- and sex-specific Recommended Dietary Allowance (RDA). Since the study relied on a single 24-hour recall, we could not apply the Estimated Average Requirement (EAR) cut-point method, which requires multiple days to account for intra-individual variation. The < 75% RDA cut-off was used as a pragmatic screening tool to identify children at high risk of inadequacy, a method consistent with established pediatric nutrition surveys in resource-limited settings.  Low serum zinc was defined as serum zinc <65 µg/dL, a cut-off recommended for all children under 10 years of age.  Overweight and obesity: For children aged 5–9 years, overweight and obesity were defined as BMI-for-age z-score (BAZ) > +1 SD and > +2 SD according to WHO growth standards. This has now been explicitly stated in the Anthropometric measurements section.  This explanation has been added to the Data collection procedures section, page 6-10. |
| Analytical rationale requires strengthening  Although statistical methods are technically sound, the rationale for the age-group comparison needs explanation:  -Why under 5 and 5–9 years?  -Was this driven by intervention design? biological differences? programmatic needs?  Also clarify whether any adjustments were considered for:  -Sex  -Socioeconomic status  -Centre location  Even if you ultimately chose descriptive analyses.  Results should be better aligned to stated objectives  -Currently, findings are presented correctly but feel disconnected due to the absence of explicit objectives. After adding objectives, ensure result sections map clearly to them. | We thank the Editor for this constructive comment regarding the analytical rationale and alignment between objectives and results.  Rationale for age-group comparison: The age stratification (<5 years vs. 5–9 years) was determined a priori based on both developmental and programmatic considerations. Children under 5 years represent the critical early-intervention period for ASD, while children aged 5–9 years correspond to the early school-age stage, characterized by different nutritional demands, feeding behaviors, and intervention goals. This classification is consistent with national ASD intervention guidelines in Vietnam and international child development frameworks. We have clarified this rationale in the revised Statistical Analysis section.  Consideration of covariates: We explored potential stratification by sex, socioeconomic status, and centre location. However, given the small sample size and the descriptive, exploratory nature of this baseline study, multivariable adjustment was not statistically appropriate. These variables are therefore presented descriptively, and this analytical decision is now explicitly stated in the Methods.  Alignment of results with objectives: Following the reviewer’s suggestion, explicit study objectives have been added to the Introduction. The Results section has been reorganized to directly correspond to each stated objective (anthropometric status, micronutrient biomarkers, dietary intake, and age-group comparisons), improving internal coherence between sections.  These revisions have been incorporated into the Introduction, Methods and Results sections (Pages 3–14). |
| Study area description is confusing  Clarify:  -Whether Nghe An province is urban/rural mixed  -Whether intervention centres are government or private  -Whether ASD diagnosis procedures differ across centres  -This contextual information is needed for interpretation. | We thank the editor for highlighting the need for clearer contextual description.  We have revised the Materials and Methods section to clarify that Nghe An province includes both urban and rural areas, that participants were recruited from private intervention centres, and that ASD diagnoses were made independently at specialized hospitals according to DSM-5 criteria. This information has been added to improve interpretability and reproducibility. |
| Minor Comments  -The manuscript is generally readable, but several sentences in the introduction and discussion can be clarified.  -Provide more detail on quality control in laboratory analyses.  -Cite Vietnamese national nutrition statistics for comparison in the results (you cite them in discussion but could bring them earlier).  -Move lengthy statistical calculations (power, precision) to supplementary files to improve flow. | We thank the Editor for these helpful suggestions.  Clarity of writing: Several sentences in the Introduction and Discussion have been revised for clarity and conciseness, with minor language edits to improve readability.  Laboratory quality control: Additional details on laboratory quality control procedures (pre-analytical, analytical, and external quality assessment) have been added to the Methods section to enhance transparency and reproducibility.  Comparison with Vietnamese national nutrition statistics: Relevant Vietnamese national nutrition statistics are now cited in the Results section (Table 3 and accompanying text) to facilitate direct comparison, rather than being discussed only in the Discussion.  Statistical calculations: Lengthy descriptions of power and precision calculations have been moved to the S1 File to improve the flow of the main manuscript.  We believe these revisions have strengthened the manuscript and improved its clarity and interpretability. |
| Reviewer #1: I propose the following enhancements to improve the manuscript:  1. The introduction lacks a clear research gap and statement of the problem or scope of study.  2. Specific objectives or research questions are missing in the introduction.  3. Predictor analysis is not adequately addressed.  4. Results should be presented in alignment with specific objectives or research questions.  5. The sufficiency of the sample size for a cross-sectional design is questionable.  6. The manuscript does not detail the sampling method employed.  7. The introduction suffers from a lack of conceptual and theoretical clarity.  8. Operational definitions are absent in the study.  9. For a community-based study, the sampling procedure needs explanation.  10. The description of the study area and sample estimation appears confusing. | We thank you for the detailed and constructive feedback. Below are our responses to each of the 10 points raised:  1. We have restructured the Introduction to explicitly state the research gap. Specifically, we highlighted that while previous studies in Vietnam focused mainly on anthropometry, there is a significant lack of integrated data linking physical growth to dietary patterns and micronutrient biomarkers, especially in regional provinces like Nghe An. (See Introduction, Page 3-4).  2. We have added four specific, numbered objectives at the end of the Introduction (Page 4) to clarify the scope of the study: (1) Assess anthropometric status; (2) Quantify micronutrient biomarkers; (3) Describe dietary intake; and (4) Compare preschool vs. school-aged children.  3. We have added a clarification in the Statistical Analysis section (Page 11). While potential predictors such as sex, socioeconomic status, and center location were considered, we ultimately prioritized descriptive and non-parametric comparative analyses to avoid model overfitting and unstable estimates given the sample size.  4. The Results section has been restructured to map directly onto the four new objectives. Section headings now follow the sequence of the objectives to ensure a logical and transparent flow of findings.  5. We acknowledge the small sample size as a limitation. However, we have justified its sufficiency for an exploratory study by providing post-hoc power calculations. Detailed calculations are available in S1 File.  6. We have clarified that the study used a census-based approach. We invited the total eligible population (100% enrollment) of children at the five participating centers during the study period.  7. We have revised the Introduction to provide a stronger conceptual framework, linking ASD-related feeding difficulties (sensory sensitivities, gastrointestinal issues) to the specific socioeconomic and medical context of Central Vietnam.  8. We have added a dedicated section for "Operational Definitions". This includes specific criteria for constipation, diarrhea, inadequate nutrient intake (<75% RDA), and age-specific thresholds for anemia, iron deficiency, and low serum zinc.  9. We have clarified the sampling procedure by describing the "Referral Pathway": children are diagnosed at specialized hospitals and then referred to private intervention centers. We invited all accessible centers in the province, and the five that provided institutional approval were included.  10. We have clarified that Nghe An is a province with mixed urban and rural districts. We also simplified the sample description by explaining that we targeted a census of all eligible children within the participating centers rather than a random sample of the entire province. |
| Reviewer #2: Good work  Thanks dear authors for your efforts and your work  Well Written manuscript and good idea for research | We sincerely thank the reviewer for the positive comments and encouraging feedback. We appreciate your time and recognition of the relevance and clarity of our work. Your support is highly valued. |
