# Supplementary material for: Micronutrient status and dietary patterns among children with autism in Central Vietnam: A cross-sectional baseline survey to inform targeted intervention
Source: PLOS Glob Public Health. 2026 May 13;6(5):e0006385. doi: 10.1371/journal.pgph.0006385 (PMC13170880; doi:10.1371/journal.pgph.0006385)
Supplement: Attachment — Submitted filename: Response_to_Reviewers_auresp_2.docx [file pgph.0006385.s008.docx]

**Micronutrient status and dietary patterns among children with autism in Central Vietnam: A cross-sectional baseline survey to inform targeted intervention**

*Manuscript Number: PGPH-D-25-01847R1*

We take this opportunity to thank the editor and reviewers of our paper for their kind collaboration to the improvement of this manuscript. We have taken intro account all the concerns raised and we have made the suggested modifications. We have implemented numerous improvements to the paper. Below we justify our replies to the suggestions made by the repected reviewers of the paper.

| Editor comments | Author(s) response |
| --- | --- |
| Strengthen the Conceptual and Analytical Framing  The study remains largely descriptive. While this is understandable given the sample size, the manuscript would benefit from a clearer conceptual structure.  Please provide a brief conceptual framing (either as a short paragraph or simple framework) linking:  -ASD-related feeding challenges  -Dietary intake patterns  -Micronutrient deficiencies  -Anthropometric outcomes  This will help position your findings within a broader scientific understanding rather than presenting them as isolated observations. | We sincerely thank the Editor for this insightful suggestion. We agree that a conceptual structure enhances the manuscript by moving beyond descriptive observations to a more integrated understanding of the health challenges in children with ASD.  In response, we have developed and included a Mechanistic Conceptual Framework (now Figure 1) and added a corresponding explanatory paragraph in the Methods section (pages 4-5, lines 73-91). |
| Deepen the Discussion and Interpretation  The discussion currently focuses primarily on describing findings rather than interpreting them.  Please strengthen this section by:  -Providing more explanation of why the observed patterns may occur,  -Reflecting on contextual drivers (e.g., feeding behaviour, caregiver constraints, food environment),  -Linking findings more explicitly to existing literature and mechanisms. | We sincerely thank the Editor for this insightful feedback. We have significantly revised the Discussion (Pages 18–20) to move beyond descriptive reporting and focus on the mechanistic interpretation of our findings.  We have addressed this by:  We have expanded the Discussion to explain the underlying reasons for the high prevalence of stunting and micronutrient deficiencies. Specifically, we now elaborate on how the sensory-driven food aversions (hypersensitivity to textures and odors) lead to a "restrictive dietary phenotype." We also clarify that the observed "linear growth retardation" (stunting) in our cohort is a chronic manifestation of long-term caloric and micronutrient insufficiency, rather than acute malnutrition. (See Discussion, Paragraph 2-4)  We have strengthened the reflection on contextual drivers unique to Central Vietnam. We added a detailed analysis of how low household income (72.9% of the cohort) acts as a critical moderator, forcing caregivers to rely on affordable but nutrient-poor staples. We also discussed the "caregiver burden," where the intensive effort required to manage ASD-related feeding challenges often leads to a compromised food environment at home. (See Discussion, Paragraph 5).  We have integrated a "Mechanistic Framework" (now illustrated in Fig 1) to connect behavioral traits (food selectivity) with physiological outcomes (zinc/iron deficiency and GI distress). We have also replaced several descriptive citations with high-impact systematic reviews and meta-analyses (e.g., Sharp et al., 2013; McElhanon et al., 2014) to ground our findings in established global ASD nutritional science. (See Discussion, Paragraph 3 & 6). |
| Expand the Limitations Section  While some limitations are acknowledged, this section should be strengthened.  In particular, please explicitly discuss:  -The implications of convenience-based centre selection,  -The small sample size (n = 48) and its impact on inference,  -The lack of multivariable analysis, and how this affects interpretation,  -The limited generalisability of findings beyond the study setting. | We appreciate the Editor’s rigorous evaluation of our methodology. We have significantly expanded the Discussion section (Pages 20-21, Lines 363-376) to provide a more transparent and critical assessment of the study's constraints.  We have specifically addressed the following points:  We have explicitly discussed how selecting participants from specialized intervention centers may introduce selection bias. We acknowledge that families accessing these services might have different socioeconomic backgrounds or higher levels of caregiver engagement compared to the general ASD population in Vietnam.  We have addressed the impact of our sample size (n = 48) on statistical power. We now clarify that while the sample size is sufficient for a descriptive baseline assessment, it increases the margin of error and limits the ability to detect subtle associations or subgroup differences.  We have added a discussion on the absence of multivariable analysis. We acknowledge that without adjusting for confounders such as ASD severity or specific household demographics, we cannot establish definitive causal relationships. We have tempered our conclusions to reflect the exploratory nature of this analysis.  We have explicitly stated that our findings may not be generalizable to children with ASD across all of Vietnam or in different socioeconomic settings. We now present this study as a "critical screening phase" and a "context-specific foundation" rather than a nationally representative profile.  (See Discussion, Paragraph 7). |
| Minor Clarity Improvements  -Review the manuscript for any remaining minor language inconsistencies.  -Ensure that all sections maintain clarity and conciseness, particularly in the discussion.  Conclusion  This is a valuable and relevant study addressing an important gap in the literature. The dataset is rich, and the revisions have significantly improved the manuscript. With further strengthening of the conceptual framing, interpretation, and limitations, the manuscript will be well-positioned for publication. I encourage you to address the points above in your next revision. | We are encouraged by your positive assessment of our study and the recognition of our dataset's value. We have conducted a final, comprehensive review of the manuscript to ensure maximum clarity, conciseness, and linguistic consistency.  The entire manuscript has been thoroughly proofread. We have standardized all terminology (e.g., ensuring "behavioral" and "centers" follow American English conventions throughout) and corrected minor grammatical errors. We have also ensured that all abbreviations are defined upon their first mention.  We have streamlined the Discussion section by removing redundant data repetitions and focusing on the interpretive "mechanistic pathway." We have used active voice where appropriate to improve readability and ensured that each paragraph transitions logically to the next. |
